# Supplementary material for: Identification of risk factors and establishment of prediction models for mortality risk in patients with acute kidney injury: A retrospective cohort study
Source: PLoS One. 2024 Oct 23;19(10):e0312482. doi: 10.1371/journal.pone.0312482 (PMC11498729; doi:10.1371/journal.pone.0312482)
Supplement: S1 File — (DOC) [file pone.0312482.s001.doc]

**STROBE Statement**

Checklist of items that should be included in reports of observational studies

| **Section/Topic** | Item No | Recommendation | Reported on Page No |
| --- | --- | --- | --- |
| **Title and abstract** | 1 | (*a*) Indicate the study’s design with a commonly used term in the title or the abstract | P1, line2 |
|  |  | (*b*) Provide in the abstract an informative and balanced summary of what was done and what was found | P2, line23-43 |
| Introduction | | | |
| Background/rationale | 2 | Explain the scientific background and rationale for the investigation being reported | P3-4, line46-47 |
| Objectives | 3 | State specific objectives, including any prespecified hypotheses | P4, line78-87 |
| Methods | | | |
| Study design | 4 | Present key elements of study design early in the paper | P4, line90 |
| Setting | 5 | Describe the setting, locations, and relevant dates, including periods of recruitment, exposure, follow-up, and data collection | P4, line90-96 |
| Participants | 6 | (*a*) *Cohort study*—Give the eligibility criteria, and the sources and methods of selection of participants. Describe methods of follow-up  *Case-control study*—Give the eligibility criteria, and the sources and methods of case ascertainment and control selection. Give the rationale for the choice of cases and controls  *Cross-sectional study*—Give the eligibility criteria, and the sources and methods of selection of participants | P5, line97-108 |
|  |  | (*b*) *Cohort study*—For matched studies, give matching criteria and number of exposed and unexposed  *Case-control study*—For matched studies, give matching criteria and the number of controls per case | N/A |
| Variables | 7 | Clearly define all outcomes, exposures, predictors, potential confounders, and effect modifiers. Give diagnostic criteria, if applicable | P6, line 111-127 |
| Data sources/measurement | 8* | For each variable of interest, give sources of data and details of methods of assessment (measurement). Describe comparability of assessment methods if there is more than one group | P6, line 111-127 |
| Bias | 9 | Describe any efforts to address potential sources of bias | P6, line 112 |
| Study size | 10 | Explain how the study size was arrived at | P6, line 128-129 |
| Quantitative variables | 11 | Explain how quantitative variables were handled in the analyses. If applicable, describe which groupings were chosen and why | P7, line 131-150 |
| Statistical methods | 12 | (*a*) Describe all statistical methods, including those used to control for confounding | P7, line 131-150 |
|  |  | (*b*) Describe any methods used to examine subgroups and interactions | P7, line 131-150 |
|  |  | (*c*) Explain how missing data were addressed | P6, line 112 |
|  |  | (*d*) *Cohort study*—If applicable, explain how loss to follow-up was addressed  *Case-control study*—If applicable, explain how matching of cases and controls was addressed  *Cross-sectional study*—If applicable, describe analytical methods taking account of sampling strategy | P7, line 131-150 |
|  |  | (*e*) Describe any sensitivity analyses | P7, line 131-150 |

| **Section/Topic** | Item No | Recommendation | Reported on Page No |
| --- | --- | --- | --- |
| Results | | | |
| Participants | 13* | (a) Report numbers of individuals at each stage of study—eg numbers potentially eligible, examined for eligibility, confirmed eligible, included in the study, completing follow-up, and analysed | P7, line 154 |
|  |  | (b) Give reasons for non-participation at each stage | P7, line 154 |
|  |  | (c) Consider use of a flow diagram | P7, line 154 |
| Descriptive data | 14* | (a) Give characteristics of study participants (eg demographic, clinical, social) and information on exposures and potential confounders | P8, line 155-174 |
|  |  | (b) Indicate number of participants with missing data for each variable of interest | p8, line 155-174 |
|  |  | (c) *Cohort study*—Summarise follow-up time (eg, average and total amount) | P8, line 155-158 |
| Outcome data | 15* | *Cohort study*—Report numbers of outcome events or summary measures over time | P8, line 155,165 |
|  |  | *Case-control study—*Report numbers in each exposure category, or summary measures of exposure | NA |
|  |  | *Cross-sectional study—*Report numbers of outcome events or summary measures | NA |
| Main results | 16 | (*a*) Give unadjusted estimates and, if applicable, confounder-adjusted estimates and their precision (eg, 95% confidence interval). Make clear which confounders were adjusted for and why they were included | P8-10, line 176-207 |
|  |  | (*b*) Report category boundaries when continuous variables were categorized | P9-10, line 209-216 |
|  |  | (*c*) If relevant, consider translating estimates of relative risk into absolute risk for a meaningful time period | P9-10, line 209-216 |
| Other analyses | 17 | Report other analyses done—eg analyses of subgroups and interactions, and sensitivity analyses | P10-11, line 219-237 |
| Discussion | | | |
| Key results | 18 | Summarise key results with reference to study objectives | P11-12, lin3 239-245 |
| Limitations | 19 | Discuss limitations of the study, taking into account sources of potential bias or imprecision. Discuss both direction and magnitude of any potential bias | P15-16, line 321-332 |
| Interpretation | 20 | Give a cautious overall interpretation of results considering objectives, limitations, multiplicity of analyses, results from similar studies, and other relevant evidence | P12-16, line 246-307 |
| Generalisability | 21 | Discuss the generalisability (external validity) of the study results | P14-15, line 308-320 |
| Other Information | | | |
| Funding | 22 | Give the source of funding and the role of the funders for the present study and, if applicable, for the original study on which the present article is based | P16, line 347-348 |

**Give information separately for cases and controls in case-control studies and, if applicable, for exposed and unexposed groups in cohort and cross-sectional studies.*

**Note:** An Explanation and Elaboration article discusses each checklist item and gives methodological background and published examples of transparent reporting. The STROBE checklist is best used in conjunction with this article (freely available on the Web sites of PLoS Medicine at http://www.plosmedicine.org/, Annals of Internal Medicine at http://www.annals.org/, and Epidemiology at http://www.epidem.com/). Information on the STROBE Initiative is available at www.strobe-statement.org.
